# The influence of health literacy and knowledge about smoking hazards on the intention to quit smoking and its intensity: an empirical study based on the data of China’s health literacy investigation

**DOI:** 10.1186/s12889-023-17292-1

**Published:** 2023-11-28

**Authors:** Siwen Sun, Huifang Yu, Jie Ling, Dingming Yao, Haixiao Chen, Guilin Liu

**Affiliations:** 1 Taizhou Hospital of Zhejiang Province affiliated to Wenzhou Medical University, Taizhou, Zhejiang China; 2 School of Public Health and Management, Wenzhou Medical University, Wenzhou, Zhejiang China; 3 Jiaxing Center for Disease Control and Prevention, Jiaxing, Zhejiang China; 4 Zhejiang Provincial Center for Disease Control and Prevention, Hangzhou, Zhejiang China

**Keywords:** Health literacy, Knowledge about Smoking hazards, Intention to quit Smoking, Intensity of intention

## Abstract

**Objective:**

This study explored the relationship between smokers’ health literacy, knowledge of smoking hazards, and their intention to quit.

**Methods:**

Based on data from the 2019 Health Literacy and Tobacco Use Surveillance among residents of a city in Zhejiang Province, 1120 male smokers were screened. Differential tests were used to analyze whether smokers with varying levels of health literacy and knowledge about smoking hazards differed in their intention to quit smoking and the intensity of their intention. A multi-factor logistic regression model was constructed to explore the extent of these differences.

**Results:**

Only 24.8% of smokers had higher health literacy. Among smokers, those with an intention to quit had a higher health literacy level compared to those without such intention (32.7% vs. 17.0%, *p* < 0.001). Health literacy levels did not differ significantly between groups with different intensity of intention to quit (34.2% vs. 31.9% vs. 30.1%, *p* = 0.435). About 48.7% of the smokers a higher level of knowledge about smoking hazards. It was more prevalent in the intent to quit group compared to the no intent to quit group (54.0% vs. 43.4%, *p* < 0.001), and the low intent to quit group had lower knowledge compared to the moderate and high intent to quit groups (49.1% vs. 56.6% vs. 63.4%, *p* = 0.011). After adjusting for other influences, smokers with lower health literacy were less likely to have intention to quit (OR = 0.659, *p* = 0.016). And the association between knowledge about smoking hazards and whether smokers have the intention to quit is no longer significant, but it significantly affects the intensity of the intention to quit among smokers who already have the intention (OR = 0.623, *p* = 0.005).

**Conclusion:**

General health literacy may play a role in facilitating smokers’ progression from the stage of no intent to quit to one of intent to quit, but a more specific understanding of the harms of smoking may be needed to increase the strength of intent to quit.

## Background

Smoking is a globally recognized health-risk behavior that causes death, disease, and disability. In 2019, 7.69 million deaths and 200 million disability-adjusted life years (DALYs) were attributed to tobacco use, with men accounting for 80% of smoking-attributable deaths [1]. In the 2019 Global Burden of Disease study, smoking was ranked the top risk factor of mortality in men [2]. Tobacco use is even more severe in China, where the 2018 China Adult Tobacco Survey (GATS) results showed that the adult smoking rate in China was 26.6% and the number of smokers was as high as 308 million [3]. Tobacco use is a public health issue that must be closely monitored, both in China and worldwide.

Motivating smokers to quit smoking is important for reducing the prevalence of smoking and the associated harm. In China, the intention to quit smoking remains low among current smokers, with only 16.1% of smokers planning to quit or considering quitting within the next 12 months according to a 2018 national survey [3]. Theories related to behavior change suggest that the intention to change is an important stage in the behavioral change process [4, 5]. The lack of intention to quit is one of the major barriers to smoking cessation among Chinese smokers, and identifying the factors that affect the intention to quit is important for tobacco control. In addition to demographic factors, such as gender [6], age [7], race [8], education [9], and employment [10], social factors, such as smoke-free legislation [11], family smoking restraints [12], and other related factors, such as nicotine dependence [13], advice from doctors [14], and media interventions [15] may be influential factors in the intention to quit. Although current studies have addressed an increasing number of factors relating to the intention to quit smoking, few have examined health literacy and smoking hazard knowledge as independent influences on the intention to quit smoking.

Health literacy is defined as the skills that enable individuals to obtain, understand, appraise, and use information to make decisions and take actions that will have an impact on health status [16]. Lower health literacy is an important risk factor that can compromise health behaviors and outcomes, such as daily exercise [17, 18], dietary habits [19], chronic diseases [20], and self-rated health status [21]. The Chinese Citizen Health Literacy Questionnaire is used to measure health literacy in China. In 2020, the average health literacy level of Chinese residents was 23.15%, and more than 3/4 of the residents had lower health literacy [22]. The lack of health literacy is particularly prevalent among lower social classes [23], and low education levels, low income, and low occupational status are characteristics not only of most people who lack health literacy, but also of many smokers, meaning that low health literacy levels and higher smoking rates often coexist in the same population. Previous research has demonstrated a significant association between health literacy and smoking-related behaviors and outcomes. Smokers with better health literacy were more likely to attempt to quit [24] and were significantly less likely to relapse following cessation treatment [25]. Although there is limited research on the direct association between health literacy and the intention to quit, available studies have demonstrated an association between health literacy and factors that influence the intention to quit. For example, Stewart demonstrated significant associations between health literacy and nicotine dependence, smoking risk perceptions [26]. Furthermore, nicotine dependence and smoking risk perceptions have been identified as important influences on the intention to quit [27, 28]. Therefore, it is reasonable to believe that health literacy may significantly influence the intention to quit smoking. Of note, there is only one smoking-related question in the questionnaire commonly used to measure health literacy in China. Hence, this study added knowledge of smoking hazards as a measure of smokers’ awareness of smoking hazards. He et al. found that smoking hazard knowledge was related to the intention to quit [14], but that study did not examine the strength of the intention to quit.

Measures of behavioral intention usually include a valence and an extremity, with valence being used to distinguish between intenders and non-intenders, and an extremity distinguishing between the strength of intention (i.e., general intention and strong intention) [29, 30]. Behavioral intention is an important predictor of behaviors, but many researchers have found that intention does not explain all the differences in behaviors. This is referred to as the “intention-behavior gap” [29, 31]. The strength of intention can influence this gap to some extent, and a stronger intention is usually a better predictor of behavioral change which tends to narrow the intention-behavior gap. To study the intention to quit smoking, we should consider not only whether smokers intend to quit but also the intensity of their intention to quit. This study aimed to explore the effects of health literacy and knowledge of smoking hazards on the intention to quit smoking and its intensity. Identifying additional intervening factors associated with the intention to quit is important for the development and implementation of effective cessation measures.

## Methods

### Respondent

This study included smokers, aged 15–69 years, in a city of Zhejiang Province in the 2019 Health Literacy and Tobacco Use Surveillance. Only male smokers were included in the analysis because the female smokers’ sample size was too small, with only six female smokers. The survey was conducted from May to September 2019. Permanent residents who had lived at the survey site for a cumulative period of more than six months during the year prior to the start of the survey were included, except for those who lived collectively in hospitals, dormitory rooms, nursing homes, and other institutional places.

### Sampling method

This survey was initially conducted as part of the Health Literacy Monitoring Program of Zhejiang Province, and its main purpose was to investigate the health literacy rate of the residents. According to the formula $n = \frac{{{z_{\alpha /2}}^2 * p * (1 - p)}}{{{\delta ^2}}} * {\text{deff}}$, which includes α = 0.05, *p* = 24.39% as the city’s residents’ health literacy rate in 2018, δ = 0.02, and deff = 2, it can be inferred that *n* = 3544. Considering the requirement of a sample size of not less than 640 people per county as stipulated in the Health Literacy Monitoring Program of Zhejiang Province and the possibility of the existence of invalid questionnaires, we finally decided to collect 680 samples per county. There are seven counties in the city and the total sample size is 4760. Using a multi-stage stratified sampling method, four streets (or townships) were randomly selected from each of the city’s seven counties, two communities (or villages) were selected for each sample street (or township), 85 households were selected for each community (or village), and one residential population, aged 15–69 years, was selected for each household as the survey target. If there is a refusal to take the survey, other households in that community (or village) will be selected to fill in until 85 are completed. After excluding 50 smokers who did not know whether they intended to quit smoking, 1120 male current smokers (hereafter, referred to as smokers) were included in the analysis sample.

### Statistical method

Statistical analysis was performed using SPSS, version 22.0 (IBM Corp., Armonk, NY, USA). The basic characteristics of the samples were expressed as composition ratios (%), as all variables were categorical. The chi-square test was used to test for differences between groups of dichotomous variables, and the Mann-Whitney U test or Kruskal-Wallis H test was used to test for differences between groups of ordered categorical variables. Dichotomous and ordered logistic regression models were used to analyze the effects of health literacy and knowledge of smoking hazards on the intention to quit and the intensity of the intention to quit smoking, respectively. The test level for each hypothesis was set at α = 0.05.

### Questionnaire content and assignment criteria

The questionnaire included basic demographic data, health literacy information, and the tobacco use survey. The tobacco use survey included current smoking status, knowledge of smoking hazards, and the intention to quit smoking. The health literacy of smokers was investigated using the National Health Literacy Questionnaire, prepared by the National Health Commission of the People’s Republic of China, which was designed by public health, health education, and clinical medicine experts. The questionnaire has an overall Cronbach’s alpha of 0.95 and a Spearman-Brown coefficient of 0.94 [32]. The total score of the questionnaire was 66 points. A score of 80% (53 points) or more of the total score was considered high health literacy, and a score of less than 53 points was considered low health literacy [33]. Questions on knowledge of smoking hazards included the diseases caused by smoking and secondhand smoke. The intention to quit smoking was divided into two categories: those who currently intended to quit smoking and those who did not intend to quit smoking currently. To further explore the factors influencing the intensity of the intention to quit, smokers with the intention to quit were divided into three levels of intention intensity according to the length of time until cessation. The specific variable names and assignment criteria are shown in Table 1.

**Table 1:** Variable definitions and assignment criteria

Variable	Definition and assignment
Health literacy	1=“score ≥ 53”;0=“score < 53”
Knowledge of smoking hazards	1=“score = 6”;0=“score < 6”
Smoking can cause stroke	1=“yes”;0=“no/not sure” the total score for each question is the score of the respondents’ knowledge of smoking hazards, with a score range of 0–6
Smoking can cause heart disease	
Smoking can cause lung cancer	
Secondhand smoke can cause heart disease in adults	
Secondhand smoke can cause lung disease in children	
Secondhand smoke can cause lung cancer in adults	
Intention to quit smoking	1="Ready to quit smoking within a month/Ready to quit smoking within 12 months/Will quit smoking, but not within 12 months”;0= “Don’t want to quit smoking”
Intensity of intention to quit smoking	1=“Will quit smoking, but not within 12 months”;2=“Ready to quit smoking within 12 months”3= “Ready to quit smoking within a month”

## Results

### General respondent characteristics

Of the 1120 male smokers, the middle-aged group, aged 40–59 years, accounted for the largest proportion (52.3%), followed by the elderly group, aged 60–69 years (28%), and the lowest proportion was the 18–39 years age group (19.6%). The education level of most respondents was junior high school and below, accounting for 69.2% of all smokers. Most smokers worked as farmers, laborers, and other manual workers, accounting for 57.6%. Rural smokers accounted for 57.9% and a significant proportion of smokers were married (95.7%). 69.1% of smokers think they are healthy. Referring to previous studies that categorized smokers’ smoking levels [34], it was found that 55.8% of smokers had a moderate/heavy daily smoking level (> 10 cigarettes per day). Of all 1120 smokers, 24.8% demonstrated a high health literacy and 48.7% scored 6 points on the knowledge of smoking hazards. Moreover, there were significant differences in smokers’ knowledge of different smoking hazards. Smokers were least aware that smoking causes stroke and heart disease, and that secondhand smoke causes heart disease in adults, with knowledge rates of 63.4%, 59.5%, and 66.6%, respectively. Conversely, approximately 90% of the smokers were aware that smoking and secondhand smoke can cause lung cancer in adults and lung disease in children.

A total of 49.64% of all smokers had an intention to quit, and the population characteristics were different between the group with and without an intention to quit. Compared to the group with no intention to quit, the group with intention to quit had a higher proportion of smokers under 60 years of age, with a high school education or higher, engaged in non-manual labor occupations, and classified as non-daily(occasional smokers) or light-daily smokers( ≦ 10 cigarettes per day). The proportion of smokers with higher health literacy and knowledge of smoking hazards was greater in the group intending to quit compared to the group with no intention to quit (32.7% vs. 17.0%, *p* < 0.001;54.0% vs. 43.4%, *p* < 0.001). No significant differences were observed among individuals with different intentions to quit smoking in terms of residential location (urban or rural), marital status, and self-assessed health status in this sample (Table 2).

**Table 2:** General respondent characteristics

Characteristics	Total sample (%, *n* = 1120)	No intention to quit smoking (%, *n* = 564)	Intention to quit smoking (%, *n* = 556)	*p*-value
**Age(years)**				
18–39	19.6	10.5	29	< 0.001
40–59	52.3	53.9	50.7	
60–69	28	35.6	20.3	
**Education level**				
Illiterate or elementary school	31.3	36.7	25.7	< 0.001
Junior high school	37.9	40.6	35.1	
High school	16	13.5	18.5	
College or above	14.9	9.2	20.7	
**Occupation**				
Agriculture	26.9	30.7	23	< 0.001
Factory or manual	30.7	32.6	28.8	
Office, student, or other non-manual	17.9	13.1	22.7	
Public sectors	9	5.9	12.2	
Other	15.5	17.7	13.3	
**Region**				
Rural	57.9	60.6	55.2	0.066
Urban	42.1	39.4	44.8	
**Marital status**				
Not married	4.3	5	3.6	0.259
Married	95.7	95	96.4	
**Self-reported good health**				
No	30.9	31	30.8	0.921
Yes	69.1	69	69.2	
**Smoking level**				
Non-daily	10.8	6.6	15.1	< 0.001
Light daily	33.4	27.7	39.2	
Moderate/Heavy daily	55.8	65.8	45.7	
**Health literacy**				
Lower	75.2	83	67.3	< 0.001
Higher	24.8	17	32.7	
**Knowledge of smoking hazards**				
Lower	51.3	56.6	46	< 0.001
Higher	48.7	43.4	54	
Conditions smoking causes*				
Stroke				
No	36.6	43.4	29.7	< 0.001
Yes	63.4	56.6	70.3	
Heart attack				
No	40.5	46.5	34.5	< 0.001
Yes	59.5	53.5	65.5	
Lung cancer				
No	8.9	13.5	4.3	< 0.001
Yes	91.1	86.5	95.7	
Conditions secondhand smoke causes*				
Heart diseases in adults				
No	33.4	39.4	27.3	< 0.001
Yes	66.6	60.6	72.7	
Lung illnesses in children				
No	11.9	16.3	7.4	< 0.001
Yes	88.1	83.7	92.6	
Lung cancer in adults				
No	11.9	16.5	7.2	< 0.001
Yes	88.1	83.5	92.8	

*"Conditions smoking causes” and “Conditions secondhand smoke causes” were the two components of “Knowledge of smoking hazards” that were combined as “Knowledge of smoking hazards” in subsequent regression analyses

### The effects of health literacy and knowledge of smoking hazards on the intention to quit Smoking

Multifactorial binary logistic regression analysis was used to understand the relationship between health literacy, knowledge of smoking hazards, and intention to quit smoking. The regression model was constructed using health literacy and knowledge of smoking hazards as independent variables and intention to quit as dependent variables, while controlling for age, education, occupation, and smoking level, which are confounding factors that may affect smokers’ intention to quit. The model passed the Hosmer - Lemeshow Test (*p* = 0.752).

The regression model results in Table 3 showed that there was a significant association between health literacy and the intention to quit. After controlling for other factors, smokers with low health literacy were less likely to have intention than those with high health literacy (OR = 0.659, *p* = 0.016), but the effect of knowledge of smoking hazards on intention to quit was no longer significant (OR = 0.780, *p* = 0.057).

**Table 3:** Logistic regression analysis of the intention to quit smoking(*n* = 1120)

Variable	Odds ratio (95%CI)	*p*-value
**Health literacy**		
Lower	0.659(0.469–0.925)	0.016
Higher	1(ref)	
**Knowledge of smoking hazards**		
Lower	0.780(0.605–1.007)	0.057
Higher	1(ref)	
**Age**		
18~39	3.529(2.206–5.645)	< 0.001
40~59	1.631(1.186–2.241)	0.003
60~69	1(ref)	
**Education level**		
Illiterate or elementary school	1.160(0.654–2.057)	0.612
Middle school	1.009(0.606–1.681)	0.972
High school	1.020(0.623–1.672)	0.936
College or above	1(ref)	
**Occupation**		
Agriculture	1.308(0.872–1.962)	0.194
Factory or manual	1.225(0.829–1.808)	0.308
Office, student, or non-manual	1.418(0.886–2.269)	0.146
Public sectors	1.632(0.916–2.907)	0.097
Other	1(ref)	
**Smoking level**		
Non-daily	2.851(1.842–4.415)	< 0.001
Light daily	1.656(1.254–2.186)	< 0.001
Moderate/Heavy daily	1(ref)	
Hosmer and Lemeshow Test	*p* = 0.752	

### Differences in the intensity of the intention to quit Smoking among different populations

The intention to quit can be further classified into different intensities depending on when the individual plans to quit. In this study, the intention to quit smoking was used as an ordered categorical variable, and the Mann-Whitney U or Kruskal-Wallis H test was used to compare whether the intensity of the intention to quit smoking differed across groups with different health literacy levels.

**Table 4:** Differential analysis of the intensity of the intention to quit smoking

Characteristics	Intensity of intention to quit smoking					
	> 12month	≤ 12month	≤ 1month	Mean Rank	Z/H	*p*-value
	(%, *n* = 281)	(%, *n* = 182)	(%, *n* = 93)			
**Heath literacy**						
Lower	65.8	68.1	69.9	281.89	-0.781	0.435
Higher	34.2	31.9	30.1	271.54		
**Knowledge of smoking hazards**						
Lower	50.9	43.4	36.6	261.45	-2.536	0.011
Higher	49.1	56.6	63.4	293.05		
**Age**						
18~39	30.2	28.0	26.9	271.63	0.675	0.714
40~59	49.5	50.5	54.8	283.26		
60~69	20.3	21.4	18.3	276.41		
**Education level**						
Illiterate or elementary school	20.6	31.3	30.1	305.53	7.832	0.050
Middle school	35.9	32.4	37.6	277.27		
High school	20.3	16.5	17.2	265.75		
College or above	23.1	19.8	15.1	258.39		
**Occupation**						
Agriculture	22.4	26.4	18.3	276.82	2.360	0.670
Factory or manual	27.4	28.6	33.3	287.73		
Office, student, or non-manual	24.9	21.4	18.3	262.44		
Public sectors	11.7	12.6	12.9	284.42		
Other	13.5	11.0	17.2	283.35		
**Region**						
Rural	52.3	62.1	50.5	282.7	-0.751	0.453
Urban	47.7	37.9	49.5	273.32		
**Marital status**						
Not married	2.5	6.0	2.2	305.23	-0.831	0.406
Married	97.5	94.0	97.8	277.5		
**Self-reported good health**						
No	31.3	28.0	34.4	279.1	-0.064	0.949
Yes	68.7	72.0	65.6	278.24		
**Smoking Level**						
Non-daily	14.6	10.4	25.8	298.79	12.851	0.002
Light daily	33.1	46.7	43.0	298.97		
Moderate/ Heavy daily	52.3	42.9	31.2	254.22		

The results in Table 4 show that the intensity of the intention to quit did not differ significantly among those with different levels of health literacy (Z=-0.781, *p* = 0.435). However, smokers who did not answer all six smoking hazard questions correctly had a significantly lower intensity of intention to quit than smokers who answered all six questions correctly (Z=-2.536, *p* = 0.011). Moreover, there were significant differences in the intensity of the intention to quit those with different educational levels (H = 7.832, *p* = 0.050) and smoking level (H = 12.851, *p* = 0.002).

### The effect of knowledge of smoking hazards on the intensity of the intention to quit Smoking

To further validate the associations between the knowledge of smoking harm and the intention to quit found in Table 4, this study used a multifactorial ordered logistic regression analysis with knowledge of smoking hazards as the independent variable and intention to quit as the dependent variable, while controlling for educational levels and smoking level, which may have an effect on the intensity of the intention to quit (Table 5).

**Table 5:** Ordinal logistic regression analysis of the intensity of the intention to quit smoking(n = 556)

Variable	Odds ratio	95%CI	*p*-value
**Knowledge of smoking hazards**			
Lower	0.623	0.448–0.867	0.005
Higher	1(ref)		
**Educational level**			
Illiterate or elementary school	2.440	1.493–3.987	< 0.001
Middle school	1.704	1.073–2.710	0.024
High school	1.230	0.728–2.077	0.439
College or above	1(ref)		
**Smoking Level**			
Non-daily	2.059	1.276–3.320	0.003
Light Daily	1.980	1.381–2.835	< 0.001
Moderate/Heavy Daily	1(ref)		
Model Fitting Information	*p* < 0.001		
Test of Parallel Lines	*p* = 0.070		

Parallelism test results showed that the model satisfied parallelism (*p* = 0.070), and thus, could be analyzed using ordered logistic regression. The results of the likelihood ratio test show that the overall model is statistically significant. After controlling for educational level and smoking level, the intensity of the intention to quit was still lower among smokers with partial knowledge of smoking hazards than those with complete knowledge (OR = 0.623, *p* = 0.005). We also found that smokers with no schooling or only primary schooling (OR = 2.440, *p* < 0.001) and middle school (OR = 1.704, *p* = 0.024) had a higher intention to quit than those with college or higher education experience. Moreover, the smoking level was found to have a significant impact on smokers’ intentions to quit. Specifically, non-daily smokers (OR = 2.059, *p* = 0.003) and light daily smokers (OR = 1.980, *p* < 0.001) demonstrated stronger intentions to quit smoking as compared to moderate/heavy daily smokers.

## Discussion

This study explored the relationship between smokers’ health literacy, knowledge of smoking hazards, and their intention to quit. Our findings suggest that smokers with lower health literacy were less likely to have a desire to quit smoking (OR = 0.659, *p* = 0.016). There was no significant association observed between health literacy and the intensity of their intention to quit smoking. Knowledge of the hazards of smoking was significantly higher in the group with intent to quit than in the group without intent to quit. Nonetheless, after adjusting for other confounding factors, the previously observed significant effect of knowledge on smokers’ intention to quit became statistically insignificant (OR = 0.780, *p* = 0.057). However, it did have a significant influence on the intensity of intention to quit among smokers who had the intention to quit (OR = 0.623, p = 0.005).

### Smokers with higher health literacy are more likely to have intentions to quit

As the definition of health literacy suggests, it represents the ability of smokers to access and understand health information and services, and to use them to maintain and promote their health. This study found that smokers with lower health literacy were less likely to have an intention to quit smoking (OR = 0.659, *p* = 0.015). This lack of health literacy may result in smokers not knowing where to access scientific cessation information or services. Even when they have access to these, the smokers may not understand the information correctly, or believe health rumors because of their lack of knowledge. Studies have shown that people with low health literacy are more likely to trust health information from television, social media, and friends, rather than healthcare professionals [35]. Health information from general sources, such as television and social media, tends to be of lower quality and may even be rumors, in contrast to professional advice [36, 37]. This tobacco myths may prevent smokers from understanding the dangers of smoking, thus reducing the likelihood of their intention to quit.

In addition, research has found that low health literacy can lead to low self-efficacy [38], and self-efficacy is one of the factors that influences smokers’ motivation to quit smoking [39]. This may also explain the reason why health literacy affects smoking cessation intention.

This survey found that the overall health literacy level of current smokers was low at approximately 24.8%. In particular, the health literacy level of smokers who do not have the intention to quit smoking is only 17.0%. This is lower than the average health literacy level of 30% of the city’s residents in the same year (2019). However, the health literacy level of smokers with the intention to quit smoking was higher (approximately 32.7%) than the average health literacy level of the city’s residents in the same year. These results indicate that the health literacy of smokers who do not have the will to quit smoking is generally low, and special attention should be paid to targeting this key group when delivering health education.

In the regression model, the effect of knowledge of smoking hazards on whether smokers had the intention to quit was not significant. This differs from the results of previous studies [14], which may be related to the sample characteristics and the different variables included in the study. The relationship needs to be further explored in the future.

### Knowledge of smoking hazards has a positive effect on the intensity of the intention to quit Smoking

Knowledge of the harm of smoking had a significant effect on the intensity of the intention to quit (OR = 0.623, *p* = 0.005). Health literacy influences the presence or absence of an intention to quit but does not significantly influence the intensity of the intention to quit.

This result may be due to the fact that health literacy, as an individual’s comprehensive ability to understand and apply health knowledge and services, provides a general cognitive foundation that helps people understand the importance of quitting and thus the intention to quit. However, because health literacy is comprehensive rather than specific, it has less direct relevance to cessation, and therefore, may have less impact on the intention intensity and behaviors. Smokers who want to increase their intention to quit smoking and eventually take action, need to learn more about health issues that are directly related to smoking. This knowledge must be specific and direct. Providing accurate information to help people understand the physical risks and personal and social consequences of smoking, may be helpful. In the literature, there are very few studies that directly examined the association between knowledge and the intensity of the intention to quit. However, in studies that examined attitude strength, which is similar to intention intensity, knowledge is considered a predictor [40]. Luttrell explained that knowledge is related to the ability to resist persuasion, enhanced attitude stability, and the ability to predict future behaviors based on the given attitude [40]. Knowledge can also be used to predict the strength of intentions given the high correlation between intentions and attitudes in the Theory of Planned Behavior [29].

Moreover, this study found that the public is aware that smoking and secondhand smoke cause lung cancer. However, the knowledge that smoking causes stroke and heart disease, as well as the cardiac risks of secondhand smoke among the general public is still low. This may be due to mass media focusing only on the smoking effects on lung diseases, while the effects of smoking on other diseases are much less publicized. Public awareness relating to the harmful effects of smoking should be strengthened. In particular, if some smokers are already “immune” to the message that “smoking causes lung cancer,“ a new direction to draw the attention of smokers is necessary.

This study has several limitations. First, this was a cross-sectional study, which cannot confirm the causal relationship between health literacy, the knowledge of smoking hazards, the intention to quit smoking, and the intensity of the intention. And the cessation outcomes for these 1120 smokers were also not available. In the future, further research to verify the causal relationship is warranted. Second, the scope of the knowledge of smoking hazards in this study was not comprehensive, but only covered stroke, heart disease, and lung disease. Further studies should address this by providing comprehensive knowledge on smoking, including smoking cessation services and anti-smoking regulations. Third, this study was only conducted in one city of Zhejiang Province, and the results are not necessarily applicable to other provinces. Finally, the intensity of the intention to quit smoking in this study was divided by arbitrary time-frames, which may systematically underestimate motivation to quit. This is because previous research has shown that although most smokers want and intend to quit, they lack a specific plan for when they will quit [41]. Therefore, in the future, attempts could be made to use other methods to categorize smoking cessation intentions to further validate the findings of this study.

## Conclusions

In this study, based on the 2019 Health Literacy and Tobacco Use Surveillance among residents of a city in Zhejiang Province, male current smokers were screened to determine the effects of health literacy and the knowledge of smoking harm on the intention to quit and the intensity of the intention. The study aimed to increase the intention to quit and promote smoking cessation strategies. The main findings of this study are as follows. (1) Smokers with higher health literacy are more likely to desire to quit. (2) Smokers who have a greater understanding of the dangers of smoking may have a stronger intention to quit. (3) Smokers’ knowledge of the dangers of smoking is still insufficient, especially the knowledge that smoking and secondhand smoke can cause stroke and heart diseases. Findings from this study suggest that general health literacy may play a role in facilitating smokers’ progression from the stage of no intent to quit to one of intent to quit, but a more specific understanding of the harms of smoking may be needed to increase the strength of intent to quit.

## Data Availability

(ADM) The datasets used and/or analyzed during the current study are available from the corresponding author on reasonable request.

## References

[1] Collaborators GT (2021). Spatial, temporal, and demographic patterns in prevalence of smoking Tobacco use and attributable Disease burden in 204 countries and territories, 1990–2019: a systematic analysis from the global burden of Disease Study 2019. Lancet.

[2] Global burden (2020). Of 87 risk factors in 204 countries and territories, 1990–2019: a systematic analysis for the global burden of Disease Study 2019. Lancet.

[3] China CDC. Global Adult Tobacco Survey (GATS) https://extranet.who.int/ncdsmicrodata/index.php/catalog/803(accessed 12 May 2023).

[4] Cole TK (2001). Smoking cessation in the hospitalized patient using the transtheoretical model of behavior change. Heart Lung.

[5] Godin G, Kok G (1996). The theory of planned behavior: a review of its applications to health-related behaviors. Am J Health Promot.

[6] Dai H (2021). Prevalence and factors Associated with Youth Vaping Cessation intention and quit attempts. Pediatrics.

[7] Arancini L, Borland R, Le Grande M, Mohebbi M, Dodd S, Dean OM, Berk M, McNeill A, Fong GT, Cummings KM (2021). Age as a predictor of quit attempts and quit success in smoking cessation: findings from the International Tobacco Control Four-Country survey (2002-14). Addiction.

[8] Kulak JA, Cornelius ME, Fong GT, Giovino GA (2016). Differences in quit attempts and cigarette Smoking abstinence between whites and African americans in the United States: Literature Review and results from the International Tobacco Control US Survey. Nicotine Tob Res.

[9] Broms U, Silventoinen K, Lahelma E, Koskenvuo M, Kaprio J (2004). Smoking cessation by socioeconomic status and marital status: the contribution of Smoking behavior and family background. Nicotine Tob Res.

[10] Yong HH, Siahpush M, Borland R, Li L, O’Connor RJ, Yang J, Fong GT, Yuan J (2013). Urban Chinese smokers from lower socioeconomic backgrounds face more barriers to quitting: results from the international Tobacco control-China survey. Nicotine Tob Res.

[11] Hackshaw L, McEwen A, West R, Bauld L (2010). Quit attempts in response to smoke-free legislation in England. Tob Control.

[12] Li L, Feng G, Jiang Y, Yong HH, Borland R, Fong GT (2011). Prospective predictors of quitting behaviours among adult smokers in six cities in China: findings from the International Tobacco Control (ITC) China Survey. Addiction.

[13] Lin H, Chen M, Yun Q, Zhang L, Chang C (2021). Tobacco dependence affects determinants related to quitting intention and behaviour. Sci Rep.

[14] He T, Liu L, Huang J, Li G, Guo X (2021). Health Knowledge about Smoking, role of doctors, and Self-Perceived Health: a cross-sectional study on smokers’ intentions to quit. Int J Environ Res Public Health.

[15] Bala MM, Strzeszynski L, Topor-Madry R, Cahill K (2017). Mass media interventions for smoking cessation in adults. Cochrane Database Syst Rev.

[16] Nutbeam D, Lloyd JE (2021). Understanding and responding to Health Literacy as a Social Determinant of Health. Annu Rev Public Health.

[17] Shiratsuchi D, Makizako H, Nakai Y, Taniguchi Y, Akanuma T, Yokoyama K, Matsuzaki-Kihara Y, Yoshida H (2021). Association of Health Literacy with the implementation of Exercise during the declaration of COVID-19 state of emergency among Japanese Community-Dwelling Old-Old adults. Int J Environ Res Public Health.

[18] Gibney S, Doyle G (2017). Self-rated health literacy is associated with exercise frequency among adults aged 50 + in Ireland. Eur J Public Health.

[19] Zoellner JM, Hedrick VE, You W, Chen Y, Davy BM, Porter KJ, Bailey A, Lane H, Alexander R, Estabrooks PA (2016). Effects of a behavioral and health literacy intervention to reduce sugar-sweetened beverages: a randomized-controlled trial. Int J Behav Nutr Phys Act.

[20] Fabbri M, Murad MH, Wennberg AM, Turcano P, Erwin PJ, Alahdab F, Berti A, Manemann SM, Yost KJ, Finney Rutten LJ (2020). Health Literacy and outcomes among patients with Heart Failure: a systematic review and Meta-analysis. JACC Heart Fail.

[21] Nie X, Li Y, Li C (2021). The Association between Health Literacy and self-rated health among residents of China aged 15–69 years. Am J Prev Med.

[22] Sun Yang,Wang Weicheng,Lang Ying, et al. Study on the Status and Influencing Factors of Chinese Residents’ Health Literacy. Health Education and Health Promotion. 2022;17:379–382 + 391.

[23] Stormacq C, Van den Broucke S, Wosinski J (2019). Does health literacy mediate the relationship between socioeconomic status and health disparities? Integrative review. Health Promot Int.

[24] Fawns-Ritchie C, Starr JM, Deary IJ (2018). Health literacy, cognitive ability and Smoking: a cross-sectional analysis of the English Longitudinal Study of Ageing. BMJ Open.

[25] Stewart DW, Cano MA, Correa-Fernández V (2014). Lower health literacy predicts Smoking relapse among racially/ethnically diverse smokers with low socioeconomic status. BMC Public Health.

[26] Stewart DW, Adams CE, Cano MA (2013). Associations between health literacy and established predictors of smoking cessation. Am J Public Health.

[27] Chen H, Zhao B, Li X (2021). Nicotine dependence, perceived behavioral control, descriptive quitting norms, and intentions to quit Smoking among Chinese male regular smokers. Subst Use Misuse.

[28] Tran TPT, Park J, Nguyen TNP (2022). Association between perceived harm of Tobacco and intention to quit: a cross-sectional analysis of the Vietnam Global Adult Tobacco Survey. BMC Public Health.

[29] Conner M, Norman P (2022). Understanding the intention-behavior gap: the role of intention strength. Front Psychol.

[30] Shook NJ, Fazio RH, Eiser JR (2007). Attitude generalization: similarity, valence, and extremity. J Exp Soc Psychol.

[31] Rhodes RE, Dickau L (2013). Moderators of the intention-behaviour relationship in the physical activity domain: a systematic review. Br J Sports Med.

[32] Shen M, Hu M, Liu S (2015). Assessment of the Chinese Resident Health Literacy Scale in a population-based sample in South China. BMC Public Health.

[33] Li S, Cui G, Kaminga AC, Cheng S, Xu H (2021). Associations between Health Literacy, eHealth literacy, and COVID-19-Related Health behaviors among Chinese College Students: cross-sectional online study. J Med Internet Res.

[34] Savoy E, Reitzel LR, Scheuermann TS (2014). Risk perception and intention to quit among a tri-ethnic sample of nondaily, light daily, and moderate/heavy daily smokers. Addict Behav.

[35] Chen X, Hay JL, Waters EA (2018). Health Literacy and Use and trust in Health Information. J Health Commun.

[36] Iacobelli M, Cho J, Welding K (2021). Machine-assessed tar yield marketing on cigarette packages from two cities in South Korea. Tob Induc Dis.

[37] Pollay RW, Dewhirst T (2002). The dark side of marketing seemingly light cigarettes: successful images and failed fact. Tob Control.

[38] Wang C, Lang J, Xuan L, Li X, Zhang L (2017). The effect of health literacy and self-management efficacy on the health-related quality of life of hypertensive patients in a western rural area of China: a cross-sectional study. Int J Equity Health.

[39] Gallus S, Cresci C, Rigamonti V (2023). Self-efficacy in predicting Smoking cessation: a prospective study in Italy. Tob Prev Cessat.

[40] Luttrell A, Sawicki (2020). Vanessa. Attitude strength: distinguishing predictors versus defining features. Soc Pers Psychol Compass.

[41] Herzog TA, Blagg CO (2007). Are most precontemplators contemplating Smoking cessation? Assessing the validity of the stages of change. Health Psychol.

